# Depressive rumination and heart rate variability: A pilot study on the effect of biofeedback on rumination and its physiological concomitants

**DOI:** 10.3389/fpsyt.2022.961294

**Published:** 2022-08-25

**Authors:** Andy Schumann, Nadin Helbing, Katrin Rieger, Stefanie Suttkus, Karl-Jürgen Bär

**Affiliations:** ^1^Lab for Autonomic Neuroscience, Imaging and Cognition (LANIC), Department of Psychosomatic Medicine and Psychotherapy, Jena University Hospital, Jena, Germany; ^2^Max Planck Institute for Human Cognitive and Brain Sciences, Leipzig, Germany

**Keywords:** depression, rumination, heart rate variability, pupil diameter, skin conductance

## Abstract

**Objective:**

Recent studies suggest that lower resting heart rate variability (HRV) is associated with elevated vulnerability to depressive rumination. In this study, we tested whether increases in HRV after HRV-biofeedback training are accompanied by reductions in rumination levels.

**Materials and methods:**

Sixteen patients suffering from depression completed a 6-week HRV-biofeedback training and fourteen patients completed a control condition in which there was no intervention (waitlist). The training included five sessions per week at home using a smartphone application and an ECG belt. Depressive symptoms and autonomic function at rest and during induced rumination were assessed before and after each of the two conditions. We used a well-established rumination induction task to provoke a state of pervasive rumination while recording various physiological signals simultaneously. Changes in HRV, respiration rate, skin conductance, and pupil diameter were compared between conditions and time points.

**Results:**

A significant correlation was found between resting HRV and rumination levels, both assessed at the first laboratory session (*r* = -0.43, *p* < 0.05). Induction of rumination led to an acceleration of heart rate and skin conductance increases. After biofeedback training, resting vagal HRV was increased (*p* < 0.01) and self-ratings of state anxiety (*p* < 0.05), rumination (*p* < 0.05), perceived stress (*p* < 0.05), and depressive symptoms (QIDS, BDI; both *p* < 0.05) were decreased. In the control condition, there were no changes in autonomic indices or depressive symptomatology. A significant interaction effect group x time on HRV was observed.

**Conclusion:**

Our results indicate that a smartphone-based HRV-biofeedback intervention can be applied to improve cardiovagal function and to reduce depressive symptoms including self-rated rumination tendencies.

## Introduction

Impaired mood, reduced energy, repetitive negative thinking and general loss of interest are key characteristics of depression. Depression is one of the most common diseases with a rising prevalence (1, 2). And the most relevant cause of disability worldwide (3). Depression is closely linked to heart disease, with significant clinical and economic consequences (4). Longitudinal cohort studies show that depression subsequently increases the risk of cardiovascular morbidity and mortality (5, 6).

Heart rate variability (HRV) quantifies cardiac vagal control and is a robust and independent marker of cardiac mortality. Several studies reported low vagal function in unmedicated patients (7–9). Meta-analyses by Rottenberg (10) or Kemp et al. (11) demonstrated a significant relation between depression and HRV decrease. This effect becomes larger when patients suffer comorbid from generalized anxiety disorder (12). Antidepressant treatment has been reported to further decrease vagal modulation (13, 14). In a longitudinal study, Licht et al. (15) showed that tricyclic, serotonergic as well as noradrenergic antidepressants are associated with a decrease in cardiac vagal function (15).

Perception, cognition, and emotions are closely tied to autonomic regulation in specific ways and at various levels of the neuraxis. As part of the autonomic response, heart rate accelerates when an individual is confronted with physical or psychological stress. Beat-to-beat variations of heart rate are characterized by HRV. HRV is considered a non-invasive marker of autonomic function that predicts of all-cause mortality (16). It has been shown that people with higher resting HRV exhibit effective regulation of negative affect, more adaptive emotion regulatory strategies, and more flexible emotional responding (17). The higher an individual’s HRV, the better their performance was found in response inhibition and emotion regulation tasks (18–20). Thayer (21) showed that low HRV marks increased risk to stress exposure. Thus, low parasympathetic activity is associated with deficits in stress-related behavior, high negative affect and general negative health consequences.

This seems to facilitate depressive rumination, the habit of pondering over one’s own negative thoughts and feelings. It is a central feature of depression that even remains elevated after both partial and full remission (22, 23). The amount of rumination is associated with diminished responsiveness to anti-depressant medication and cognitive therapy and rumination has been demonstrated as a crucial factor in vulnerability to depression, predicting the onset, severity, and duration of future depressive episodes (24–26). Moreover, rumination involves the repetitive focusing on one’s distress symptoms or negative emotions, and strong self-referential attention (27, 28). Thus, depressed patients find it difficult to disengage from self-focusing even though it might be irrelevant in the present moment (29, 30).

Several studies have reported that rumination and worry are associated with elevated sympathetic arousal and decreased parasympathetic heart rate modulation (31–35). After experimental induction as well as spontaneous onset of rumination, a decline in HRV was reported (36–38). In a meta-analysis, Ottaviani et al. (39) summarized that rumination and perseverative cognition are accompanied by increases in heart rate, blood pressure and cortisol levels as well as by HRV withdrawal. These results indicate that rumination is a form of chronic stress that is associated with a shift in sympathovagal balance toward sympathetic predominance.

Interestingly, the close relationship of altered heart rate regulation and rumination (36, 40) might be due to the loss of inhibitory control over important subcortical regions (41). Self-referential processing in depression was associated with abnormally increased activity of medial frontal and emotion-regulating structures (42–44). Several studies found a disconnection of the medial prefrontal cortex (45–48), especially, to limbic regions such as the amygdala and insula seem to be related to deficits in affective processing and emotional evaluation (49–52).

Heart rate variability biofeedback has been demonstrated to improve clinical symptoms in patients suffering from depression (53–57). A recent meta-analysis of randomized controlled studies including a total number of 794 participants yielded a significant medium size effect (Hedges’ g = 0.38) of HRV biofeedback on depressive symptoms (58). Physiological effects seem to be primarily mediated *via* enhanced baroreflex function and cardiovagal activity (59–61). In a previous study, we found increased resting HRV and baroreflex sensitivity after an 8-week HRV biofeedback intervention in healthy volunteers (62).

In this study, we aimed to investigate whether HRV biofeedback has a specific positive effect on rumination in depressed patients. We hypothesized a correlation of resting HRV and self-reported tendencies to engage in ruminative thoughts. After a 6-week HRV biofeedback intervention, we assumed that patients report lower levels of rumination.

## Materials and methods

### Patients

We recruited 25 patients (19 women, six men; age: 41 ± 15 years; BMI: 25.5 ± 5.5 kg/m^2^) from ambulatory care either in the psychiatric outpatient ward of the Jena University Hospital or nearby resident practitioners. All participants gave written informed consent to a protocol approved by the Ethics Committee of the medical faculty of the Friedrich-Schiller University Jena (# 5423-02/18) in accordance with the Declaration of Helsinki.

Inclusion criteria were ICD-10 diagnosis of depression, age between 18 and 55, male or female, period, ability to give written informed consent to the study, stable psychopathology and constant antidepressant treatment over a 2-week screening, minimum rating of 30 on the rumination scale RRQ, unremarkable results of physical examination, ECG, laboratory investigations. Patients were instructed to refrain from smoking, heavy meals, exercise and alcohol 2 h before laboratory session.

Patients have been diagnosed with a minor (*N* = 8), moderate (*N* = 11) or severe (*N* = 2) recurrent depressive disorder, major depression (*N* = 2) or dysthymic disorder (*N* = 2). The majority of patients were treated with one or more types of antidepressant medication, including serotonergic (*N* = 13) and noradrenergic reuptake inhibitors (*N* = 1), tricyclic and tetracyclic antidepressants (*N* = 5, *N* = 1), Atypical antidepressant (*N* = 2) and antipsychotic medication (*N* = 2). Seven patients were currently not treated with antidepressants. Two weeks before and the time during the control and intervention condition, type and dose of pharmacological treatment and the frequency of psychotherapy sessions had to remain constant. Additionally, we ensured no severe changes in daily life such as job change, relocation, vacations, or study exams took place during this period.

All 25 patients started the procedure with an initial laboratory session, before starting a 6-week control condition (waitlist) or a biofeedback intervention for 6 weeks. Two patients dropped out due to stationary admission (*N* = 1) and changes in medication status during the course of the experiment (*N* = 1). Finally, 14 patients completed the control condition, and 16 patients finished the biofeedback intervention. Seven patients first completed the control condition and then conducted the biofeedback training (not randomized cross-over design). An overview of patients’ characteristics included in the control and intervention group are given in Table 1.

**TABLE 1:** Sample characteristics.

	Control	Intervention
Men/women	*N* = 3/*N* = 11	*N* = 4/*N* = 12
Age (years)	38 ± 13	42 ± 17
BMI (kg/m^2^)	24 ± 5	25 ± 5
Smoker/Non-smoker	*N* = 1/*N* = 13	*N* = 2/*N* = 14
Years of education	11 ± 1	11 ± 1
BDI	20.2 ± 7.9	21.6 ± 10.7
QIDS	13.8 ± 3.3	13.3 ± 5.6
RRS	25.1 ± 2.7	25.3 ± 6.5

BDI, Beck depression inventory; QIDS, quick inventory of depressive symptoms; RRS, rumination response scale. All data assessed at the first session.

The psychopathological state was assessed by the Beck’s Depression Inventory (BDI-II) (63), the Quick Inventory of Depressive Symptomatology (QIDS-SR16) (64), the State-Trait Anxiety Inventory (STAI) (65), and the Perceived Stress Scale (PSS-10) (66). State rumination and current tendencies for perseverative cognition were assessed by the German version of the Rumination-Reflection Questionnaire (RRQ) (67), and the rumination response style (RRS) (28).

### Timing schedule

After the recruitment interview, a first appointment was arranged in which participants had an initial laboratory assessment. During the control condition, patients waited for 6 weeks to undergo another laboratory session (waitlist). In the intervention condition, patients were instructed on how to use the training instruments [App(s) and add-on devices] and go for a test run. The training was then conducted at home (see Biofeedback intervention). After 6 weeks the intervention ends and participants underwent laboratory investigations again. At all laboratory sessions, patients performed the rumination induction paradigm and filled out all questionnaires. Repeated sessions were scheduled individually at a similar time of day in each participant between noon and early evening (12 a.m.–5 p.m.). We have ensured beforehand that no serious events were scheduled within the period of the control condition and intervention.

### Laboratory session

Resting recordings were conducted in a supine position for 15 min. The first 5 min were not analyzed, to exclude the adjustment period to the environment. The examination room was quiet and fully shaded with a low intensity ambient light source. Additionally, participants wore headphones to be isolated from a potential surrounding noise. Through a monitor fixed over the couch a dark gray ellipse was displayed on light gray background as a fixation anchor. Room temperature was controlled to 22°C.

We used the well-established rumination induction paradigm (68, 69). Patients are instructed to think of a situation that makes them feel sad or anxious. The episode may have happened in the past or may happen in the future. They are asked to think about this situation in detail especially possible causes, consequences, and their feelings. The rumination phase lasted another 10 min while all physiological signals were continuously recorded.

### Physiological recordings

Simultaneous multi-channel recordings of autonomic function were performed at rest and during rumination using a polygraph MP150 system (BIOPAC Systems Inc., Goleta, CA, United States) at 1 kHz sampling frequency. ECG was acquired by arranging three electrodes on the chest. Abdominal and thoracic respiratory movements were recorded by two individual strain gauge transducers. Skin conductance was measured continuously by the constant voltage technique on the left hands’ palm with electrodes placed at the thenar and hypothenar eminence. Pupil size changes were assessed every 4 ms by the infrared camera system RED 250 (SensoMotoric Inc., Boston, MA, United States).

### Indices of autonomic function

Artifacts and ectopic beats in the beat-to-beat interval series (BBI) were detected and removed using an adaptive filtering technique (70). The mean heart rate HR and standard deviation of BBI (SDNN) around the mean were estimated according to the established standard procedures (71). In each BBI, systolic blood pressure (SBP) was extracted as the maximum blood pressure in one cardiac cycle. We report the mean SBP over the recording. The mean breathing rate (BR) was derived from the respiration signal. Skin conductance level (SCL) and pupil diameter were estimated by averaging the whole skin conductance signal and pupil diameter values.

### Biofeedback intervention

Participants performed a biofeedback training for 6 weeks, in order to elevate heart rate variability (HRVBF). Five sessions per week were done at home, using a smartphone and an HR belt (H10 POLAR, Polar Electro Oy, Kempele, Finland).

At the start of the intervention, the resonant frequency (RF), at which HRV is highest, was estimated in the laboratory. In the first 2 weeks participants train to breath at their individual RF as a preparation for the subsequent biofeedback of heart rate. To determine RF, participants were asked to breathe according to a given rhythm (7, 6, 5, 4.5, and 4 bpm) for 2 min each, while ECG and respiration were recorded. A visual pacer was displayed on the screen above the participants lying on the couch. The respiratory trace was used to ensure that patients followed the presented rhythm. SDNN was estimated in each 2-min segment. At the breathing rate where SDNN was highest the optimal RF was extracted (62).

From week three to six, participants were asked to concentrate on the HR-curve. Their target is to synchronize their breathing rhythm with this curve by inhaling when HR increases and exhaling when HR decreases, trying to progressively expand the amplitudes of HR oscillations.

Participants trained five times a week at home. Each session comprised a 5-min resting recording and two 11-min training blocks with a break between them. At least once a week, we got in touch with each participant to discuss problems, give advice and keep motivation high.

### Statistical analysis

The effect of rumination on autonomic function was assessed based on data of all patients acquired during the first laboratory session—before the start of the intervention or control condition. We compared autonomic indices estimated during resting state and induced rumination via a paired *t*-test. According to our hypothesis, we analyzed the relationships between resting HRV with psychopathological ratings; i.e., depressive symptoms (QIDS and BDI), perceived stress (PSS), state anxiety (STAI), and rumination levels (RRQ, RRS) also assessed at the first visit using Pearson correlation coefficients.

Differential effects of the biofeedback intervention and control condition were investigated by comparing changes of resting HRV and psychopathologic ratings before (T1) and after the intervention (T2) using a general linear model with the between-subjects factor group and the within-subject factor time. Simple effects of factor time were tested in each group. Significant differences over time in psychopathological scales and HRV were then correlated with each other in an exploratory manner.

## Results

**FIGURE 1:**
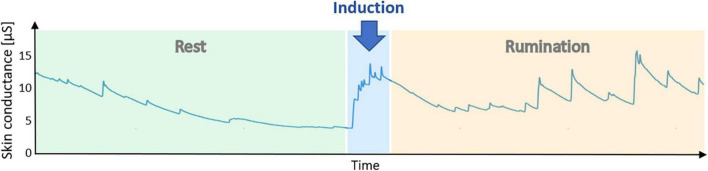
Example time course of skin conductance during a laboratory session. After 10 min of resting state, instructions to engage in rumination were displayed. The rumination phase lasted another 10 min. An elevation of skin conductance during the rumination phase indicates sympathetic activation elicited by ruminative thoughts.

Analyzing all patients at their first laboratory session, we found a significant correlation of HRV with rumination levels RRS (*r* = -0.43, *p* < 0.05). Ruminative thoughts, we than triggered during the induction paradigm. Figure 1 shows an exemplary skin conductance (SC) time course of one patient that indicates sympathetic arousal. During the resting condition there is a decreasing trend with only a few unspecific fluctuations in SC. Reading the rumination instructions already increased skin conductance substantially. Throughout the induction period, skin conductance level (SCL) remained elevated and showed multiple fluctuations that are most probably elicited by negative emotions and stress due to induced rumination.

In Table 2, all autonomic indices assessed in our study were compared between resting state and rumination induction. A paired t-test revealed significant increases in HR (*T* = 2.1, *p* < 0.05) and skin conductance levels (*T* = 2.15, *p* = 0.04).

**TABLE 2:** Changes in autonomic function during rumination compared to rest.

Parameter	Resting state	Rumination	*P*-value
HR [1/min]	75.3 ± 10.7	76.9 ± 10.8	0.046
HRV [ms]	34.5 ± 25	34.1 ± 22.4	0.850
SBP [mmHg]	129.8 ± 24.1	134.3 ± 22.6	0.076
SCL [μS]	2.1 ± 5.5	2.6 ± 6.5	0.043
BR [1/min]	12.6 ± 3.5	14.5 ± 5.1	0.107
DIA [mm]	4.5 ± 0.9	4.6 ± 0.8	0.394

HR, mean heart rate; HRV, heart rate variability; SBP, systolic blood pressure; SCL, skin conductance level; BR, breathing rate; DIA, mean pupil diameter.

**FIGURE 2:**
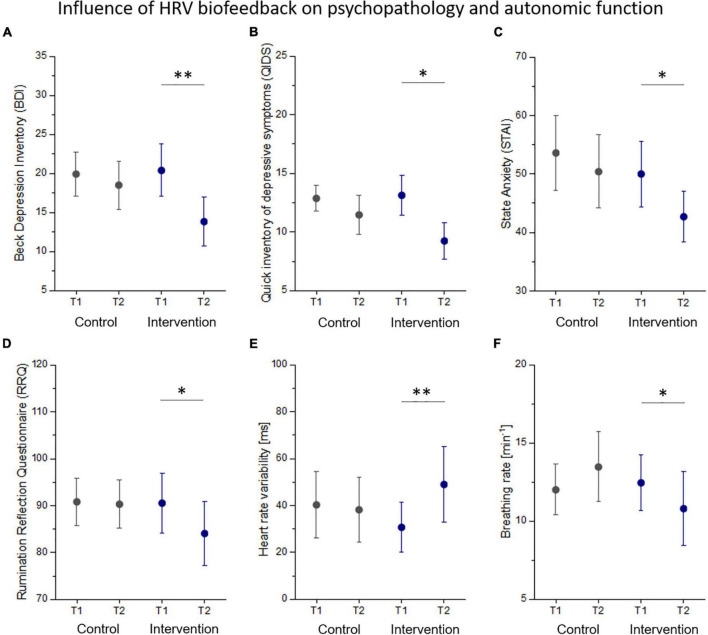
Changes in psychopathology **(A–D)** and autonomic function **(E,F)** from before (T1) to after (T2) the control and intervention condition. Statistical thresholds: *p < 0.05, ^**^p < 0.01.

Changes in autonomic function and psychopathology from before (T1) to after (T2) the control and intervention condition are listed in Table 3. There was one significant interaction effect group x time on HRV (*F* = 7.36, *p* = 0.011) that was driven by a significant increase of HRV in the intervention group (*p* = 0.005, Figure 2E). Simple effects analyses revealed that patients showed a significantly reduced breathing rate after biofeedback (*p* = 0.026, Figure 2F). Although, there was a significant reduction in self-ratings of state anxiety (STAI: *p* = 0.043, Figure 2C), rumination (RRQ: *p* = 0.032, Figure 2D), perceived stress (PSS: *p* = 0.021), and depressive symptoms (BDI: *p* = 0.001, QIDS: *p* = 0.016, Figures 2A,B) in the intervention group, we found no interaction effect on any psychopathological scale. In an exploratory approach, we correlated changes in HRV with changes in those psychopathological scales that were influenced by HRVBF and found a significant correlation between HRV and BDI (r = 0.4, *p* < 0.05).

**TABLE 3:** Changes in autonomic function and psychopathological state after biofeedback intervention and control condition.

Parameter	Control			Intervention		
	T1	T2	Significance	T1	T2	Significance
HR [1/min]	74.6 ± 10.2	73.2 ± 10	0.383	76.7 ± 12	74.6 ± 11.5	0.261
HRV [ms]	42 ± 27.6	38.2 ± 26.7	0.429	30.7 ± 20.9	49 ± 31.5	0.005
SBP [mmHg]	143.5 ± 25.4	140.2 ± 16.6	0.328	126.8 ± 24.6	122 ± 23.4	0.091
SCL [μS]	3.1 ± 8	2.3 ± 5.2	0.127	3.6 ± 7.8	3.3 ± 7.5	0.792
BR [1/min]	12 ± 3.3	12.8 ± 3.7	0.327	12.5 ± 3.5	10.8 ± 4.6	0.049
DIA [mm]	4.6 ± 0.9	4.3 ± 0.8	0.261	4.3 ± 1.0	4.0 ± 0.8	0.05
STAI-s	55.8 ± 10.9	51.1 ± 12.7	0.164	48.9 ± 9.8	41.4 ± 11.5	0.017
PSS	24.4 ± 7.2	22.6 ± 7.7	0.541	23.2 ± 9.2	16.8 ± 8.5	0.021
QIDS	13.8 ± 3.3	11.7 ± 5.3	0.129	13.3 ± 5.6	9.5 ± 5.2	0.016
BDI	20.2 ± 7.9	18.8 ± 8.7	0.536	21.6 ± 10.7	14.2 ± 10.5	0.001
RRS	25.1 ± 2.7	24 ± 4.1	0.484	25.3 ± 6.5	22.6 ± 6.3	0.057
RRQ	90.6 ± 7.7	90.1 ± 9.2	0.909	92.5 ± 10	85.0 ± 12.4	0.032

HR, mean heart rate; HRV, heart rate variability; SBP, systolic blood pressure; SCL, skin conductance level; BR, breathing rate; DIA, mean pupil diameter; STAI-s, state anxiety inventory; PSS, perceived stress scale; BDI, Beck depression inventory; QIDS, quick inventory of depressive symptoms; RRS, rumination response scale; RRQ, rumination reflection questionnaire.

## Discussion

In this study, we applied a smartphone-based HRV-biofeedback intervention over 6 weeks and assessed its influence on depressive symptoms with a special focus on rumination. We corroborated a link between resting HRV and rumination levels reported at the first visit. A rumination induction paradigm led to an accelerated heart rate and increased skin conductance when compared to rest. We found improved cardiovagal function and reduced severity of symptoms, including self-rated rumination levels after the biofeedback intervention.

Rumination has vastly adverse consequences for patients suffering from depression. As patients struggle to shift their attention away from negative self-related stimuli, those feelings determine their emotional state. The physiological arousal accompanying rumination might compromise patients’ cardiovascular health. Cardiac sympathetic activation and withdrawal of vagal HRV seem to be a consequence of rumination (69). In this study, we especially observed activation of the sympathetic nervous system during rumination, as indicated by increases in heart rate and skin conductance (72). In contrast, rumination induction did not affect HRV in our study. That was surprising since it is well-documented that experimentally and spontaneously induced rumination reduces HRV (36–38). One reason, might be that recurring negative thoughts elicit phasic heart rate reactions in a similar manner as they can be observed in the time course of skin conductance (73). Therefore, the rumination condition can hardly be considered a constant state that can be described by an HRV average over the entire phase. Additionally, it is likely that repetitive negative thoughts also occur spontaneously during the resting condition, obscuring the influence of rumination induction. Interestingly, our results corroborated the association between resting HRV and rumination as we observed a linear correlation of self-reported rumination tendencies and HRV estimated at rest. Resting HRV indicates the flexibility of the cardiac system as well as the adaptivity of cognitive processes (74).

Longitudinal studies have suggested HRV to mediate how rumination influences the progression of depressive psychopathology over time (40, 75). Increasing HRV seems to be a suitable way to alleviate depressive symptoms in the long run making *via* HRV biofeedback a valuable add-on to standard therapy (76). In a large study, HRV biofeedback led to reduced depressive symptoms over 1 year (77).

Our results suggest that an intensive biofeedback intervention over 6 weeks reduces depressive symptoms. The reduction in BDI scores was proportional to increases of HRV. Previous studies have indicated that HRV biofeedback enhances inhibitory control of the prefrontal cortex by augmenting functional brain connectivity to other regions such as the insula and amygdala (78, 79), which has a beneficial impact on emotion regulation and stress resilience (80). However, most neuroimaging studies have focused on heart rate as a target of top-down central control (78, 81). How autonomic reactions shape the experience and regulation of emotions has long been a matter of debate [see review by Pace-Schott et al. (82)]. A very recent study by Candia-Rivera et al. (83) gave experimental support to the “causation theory” by demonstrating a causal role of sympathovagal activity in the initiation (bottom-up) of emotional responses. Processing of these initiated emotions involves bidirectional communication between the heart and the brain (83). Thus, successful regulation and interpretation of physiological arousal seem to facilitate adaptive emotion regulation (84). Depressive rumination and negative affect have been linked to low interoceptive abilities that seem to be enhanced by interventions such as biofeedback (85, 86). This in turn might improve the brain-heart-connection during these emotion regulatory processes with a beneficial impact on worry, depressive symptoms, and negative affect (87).

As a main limitation of the current study, the rather small sample size needs to be highlighted. In consequence, the findings should be generalized with care. Additionally, the two groups are not well-matched with respect to age. Aging decreases resting HRV (88–90), and to reduce the effect of physical exercise on HRV (91–93). Although the difference between groups was not statistically significant, an effect of age on the effects within the groups cannot be excluded. To limit the impact of other factors, such as sex, body mass, eating, drinking, smoking, circadian rhythms, and antidepressant medication, we tried keeping conditions of the laboratory measurements as comparable as possible. However, all these factors might introduce additional variance to our statistical models. The reader has to keep in mind that we did not call patients in the control condition weekly as we did during the intervention. This social interaction and the feeling that someone cares may be also a beneficial factor for patients that is not related to biofeedback itself.

In conclusion, smartphone-based HRV biofeedback seems to alleviate depressive symptoms and self-reported rumination levels. Modern technology and smart mobile devices enable remote training, which is particularly advantageous when personal contact is limited. HRV biofeedback has even been suggested as a preventive strategy for people who exhibit an especially high psychological burden during the pandemic, such as healthcare workers, before they develop mental disorders (94). This study provides further evidence for the positive influence of HRV biofeedback on mental and cardiovascular health.

## Data availability statement

The datasets generated for this study are available on request to the corresponding author.

## Ethics statement

The studies involving human participants were reviewed and approved by Ethikkommission FSU Jena. The patients/participants provided their written informed consent to participate in this study.

## Author contributions

AS contributed to analysis and interpretation of the data and preparing the manuscript. KR and NH contributed to acquisition of the data, quality control, and preprocessing of the data. SS contributed to study conception and critical revision. K-JB contributed to study conception, preparing the manuscript, and critical revision. All authors contributed to the article and approved the submitted version.
