# Supplementary material for: Depressive rumination and heart rate variability: A pilot study on the effect of biofeedback on rumination and its physiological concomitants
Source: Front Psychiatry. 2022 Aug 25;13:961294. doi: 10.3389/fpsyt.2022.961294 (PMC9452722; doi:10.3389/fpsyt.2022.961294)
Supplement: Supplementary file 1 [file Table_1.docx]

Supplementary table 1: Additional patient characteristics (f: female; m: male; CC: control condition; IC: intervention condition).

| # | CC | IC | age | sex | diagnosis | comorbidities | Current psychotherapy |
| --- | --- | --- | --- | --- | --- | --- | --- |
| 1 | ✓ | 🗶 | 41 | f | Minor | - | Yes |
| 2 | 🗶 | ✓ | 49 | f | Minor | Adjustment disorder | Yes |
| 3 | ✓ | ✓ | 24 | f | Minor | - | No |
| 4 | ✓ | ✓ | 31 | m | Moderate | - | Yes |
| 5 | 🗶 | ✓ | 48 | f | Minor | Chronic fatigue | Yes |
| 6 | ✓ | ✓ | 21 | m | Minor | Social phobia | Yes |
| 7 | ✓ | 🗶 | 29 | m | Severe | - | Yes |
| 8 | 🗶 | ✓ | 22 | m | MDD | - | Yes |
| 9 | ✓ | ✓ | 44 | f | Dysthymia | Anxiety | Yes |
| 10 | ✓ | 🗶 | 31 | f | Minor | - | No |
| 11 | ✓ | ✓ | 65 | f | Moderate | - | Yes |
| 12 | 🗶 | ✓ | 73 | f | Minor | Adjustment disorder | Yes |
| 13 | ✓ | ✓ | 59 | f | Moderate | Anxiety | Yes |
| 14 | ✓ | 🗶 | 29 | f | Minor | Hypothyroidism | Yes |
| 15 | ✓ | 🗶 | 47 | f | Moderate | - | Yes |
| 16 | 🗶 | ✓ | 31 | f | Dysthymia | ADHD, migraine | Yes |
| 17 | 🗶 | ✓ | 41 | m | Moderate | - | Yes |
| 18 | 🗶 | ✓ | 74 | f | Moderate | Fibromyalgia | Yes |
| 19 | 🗶 | ✓ | 25 | f | Minor | - | No |
| 20 | ✓ | ✓ | 46 | f | Moderate | - | Yes |
| 21 | ✓ | 🗶 | 38 | f | Moderate | - | Yes |
| 22 | ✓ | 🗶 | 33 | f | Minor | - | No |
| 23 | 🗶 | ✓ | 44 | f | Minor | Anxiety | No |
